# The Beat

**DOI:** 10.1289/ehp.120-a306b

**Published:** 2012-08-01

**Authors:** Erin E. Dooley

## C8 Phased Out of Food Wrappers

In July 2012 the U.S. FDA released a statement that manufacturers had voluntarily ceased selling agents containing the compound C8 for food-contact use the prior summer.^^1^^ At that time, manufacturers had predicted stocks of the agents and packaging containing them would be depleted within a year. C8, or perfluorooctanoic acid, was used to keep oil and grease from seeping through food packaging. It is persistent in the environment and has been linked with testicular and kidney cancers and pregnancy-induced hypertension.^^2^^ The compound is still used in products such as cookware and clothing.

## Where’s the Cleanest Beach?

In June 2012 the Natural Resources Defense Council released its annual *Testing the Waters* report on water quality at U.S. ocean and Great Lakes beaches.^^3^^ Certain beaches in Alabama, California, Delaware, Maryland, Minnesota, New Hampshire, and Texas have exceptionally low contamination rates and strong testing and safety practices, earning them the report’s five-star rating. But the past two years had the second and third highest numbers of beach closures and advisories in the past two decades, with the majority a result of contamination with human or animal waste. The report cites measures to reduce stormwater runoff and sewage spills as top priorities for reducing beach closures and illnesses.

**Figure:**
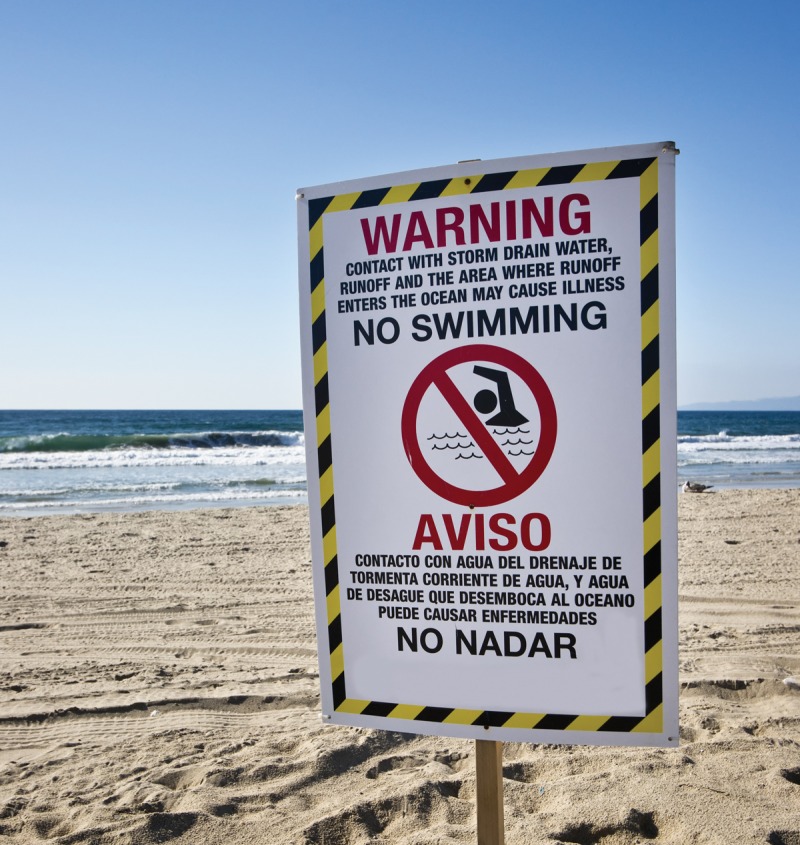
Tom Sebourn/Shutterstock

## Rio+20 Envisions *The Future We Want*

In adopting the consensus document *The Future We Want*,^^4^^ parties to the June 2012 United Nations Conference on Sustainable Development (Rio+20) renewed their commitment to a number of principles and goals supporting environmental health alongside development. Among other statements, parties expressed support for access to cleaner energy, including cleaner fossil fuels; urban planning that promotes a safe, healthy environment for all residents; improved sanitation to combat the spread of communicable diseases; sustainable food networks and water usage; and occupational settings that meet minimum safety and health standards. In conjunction with the conference, government and private stakeholders around the world declared over 700 voluntary commitments worth more than $500 billion toward meeting these goals.

## Study Refines Climate Change and Wildfire Predictions

A new study employing satellite-based wildfire data from the past decade and 16 different global climate change models (GCMs) estimates that the likelihood of fires like the summer 2011 events in western United States will particularly increase in mid- and high-latitude areas of the Earth in the next 30 years.^^5^^ For both the near- and far-term time periods assessed, models for several biomes—including Mediterranean biomes, montane grasslands and shrublands, and temperate coniferous forests—show general agreement for increased fire probability. The authors write, “Although GCMs predict that temperatures will rise virtually everywhere on Earth over the next century, future fire occurrence appears to primarily be a function of the available moisture in many areas.”

## Particles Unveiled in Flight

It has been difficult to image particulate matter (PM) without introducing artifacts caused by capturing particles for microscopic analysis. Now researchers have found a way to do so using the Linac Coherent Light Source free-electron laser, the world’s most powerful X-ray laser.^^6^^ A stream of aerosol particles was shot across the pulsed X-ray beam, producing high-resolution diffraction patterns for individual particles. Simultaneously, ion fragments ejected from the beam were analyzed with mass spectrometers to study the composition of single particles. Among other findings, airborne soot particles were shown to be more compact than previously thought, an insight that could help improve climate change models since soot’s structure determines how it scatters light.

**Figure:**
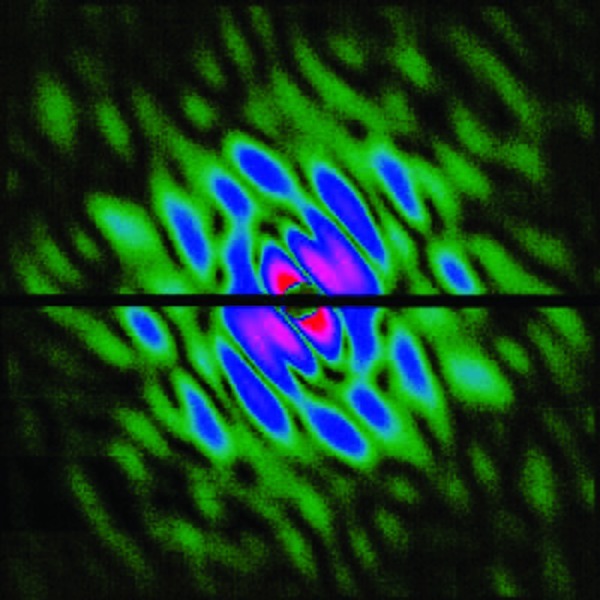
Diffraction pattern of a single soot particle. Loh et al. doi:10.1890/ES11-00345.1
